# Prostate-specific membrane antigen is undetectable in choroidal neovascular membrane

**DOI:** 10.1186/1477-3163-5-21

**Published:** 2006-08-15

**Authors:** Katyanne Dantas Godeiro, Ana Carolina de Arantes Frota, Emilia Antecka, Alexandre Nakao Odashiro, Shawn Maloney, Bruno Fernandes, Miguel Noel Burnier

**Affiliations:** 1 Department of Ophthalmology, McGill University, Montreal, QC, Canada, 3775 University Street, Room 216. Montreal, QC, H3A 2B4, Canada; 2 Department of Ophthalmology, Federal University of São Paulo, SP, Brazil, Rua Botucatu, Número 822. Vila Clementino, São Paulo, SP, 04023-062, Brazil

## Abstract

**Background:**

Choroidal neovascular membrane (CNVM) is one of the leading causes of severe visual loss and is often associated with age-related macular degeneration (AMD). Various modalities of treatment, including photocoagulation and surgery, are being considered as options, but with limited success. Prostate-specific membrane antigen (PSMA) is a type II membrane glycoprotein expressed in benign and malignant prostatic tissues, in some non-prostatic tissues, and in the endothelium of tumor-associated neovasculature of non-prostatic neoplasm. Some studies have suggested that the expression of PSMA is restricted to endothelium from tumor-associated neovasculature and might be stimulated by some tumor-secreted angiogenic factors. However, no previous study demonstrating PSMA expression in non-related tumor neovasculature, such as CNVM, has been performed to date. Furthermore, demonstration of PSMA expression in CNVM in AMD patients could reveal a novel target for antineovascular therapy. The purpose of this study was to evaluate the immunohistochemical expression of PSMA in CNVM from AMD.

**Methods:**

Immunohistochemical analysis, with a standard avidin-biotin complex technique, was performed using an anti-PSMA mouse monoclonal antibody in 30 specimens of surgically excised CNVM from AMD patients. Antibody to an endothelial cell specific marker, factor VIII, was used to confirm the location of the endothelial cells.

**Results:**

The angiogenic microvessels of the 30 cases demonstrated negative staining to PSMA while factor VIII was expressed in all cases. Seventy-five percent of the secretory-acinar epithelium of the prostatic hyperplasia specimen stained positive, confirming that the immunohistochemical technique was correctly performed.

**Conclusion:**

The absence of PSMA expression in non-tumoral neovasculature supports the theory, previously suggested, that endothelial cell PSMA expression may be stimulated by one or more tumor-secreted angiogenic factors. Angiogenesis is very important in neoplasia and the endothelial expression of PSMA in tumor-associated neovasculature may represent a target for antineovasculature-based therapy. The absence of PSMA expression in CNVM suggests that PSMA may not be a potential target for antineovasculature-based therapy.

## Background

Age-related macular degeneration (AMD) is the principal cause of registered legal blindness among those over 65 years of age in the United States, Western Europe, Australia, Canada and Japan [1-5]. The clinical hallmarks of AMD are the formation of drusen, geographic atrophy and, in advanced stage, choroidal neovascular membrane (CNVM). The mechanism of CNVM formation is growth of new vessels from the choriocapillaris through Bruch's membrane and subsequent extension into the subretinal pigment epithelium, subretinal space, or a combination of both [6-8]. Various modalities of treatment, including photocoagulation and surgery, are being considered as options, but with limited success.

Prostate-specific membrane antigen (PSMA) is a 100 kDa type II transmembrane glycoprotein, located on chromosome 11p, initially characterized by the monoclonal antibody (mAb) 7E11 [9,10]. Although PSMA exhibits in vitro neuropeptidase and folate hydrolase activity, its function in vivo has not been fully elucidated [11,12]. PSMA is strongly expressed in benign prostatic secretory-acinar epithelium, prostatic intraepithelial neoplasia, and prostatic adenocarcinoma [10,13]. PSMA expression is also described in other benign tissues, including a subset of proximal renal tubules and duodenum mucosa [13,14], and in non-prostatic malignant tumor neovasculature [15]. Studies have suggested that PSMA expression is restricted to tumor-related neovasculature and might be stimulated by tumor-secreted angiogenic factors. However, this hypothesis cannot be validated since no characterization of PSMA expression in non-related tumor neovascularization, such as CNVM, has been performed to date.

The purpose of this study was to characterize the immunohistochemical expression of PSMA in CNVM associated with AMD. The presence or absence of this protein in CNVM could contradict or support the theory that endothelial cell PSMA expression is tumor related. Furthermore, the presence of PSMA could be a new potential target for antineovascular therapy in cases of AMD.

## Materials and methods

Thirty surgically excised CNVM samples obtained at vitrectomy from patients with AMD were collected from the archives of the Henry C. Witelson Ocular Pathology Laboratory and Registry, McGill University, Montreal, Canada. Each specimen was formalin-fixed and paraffin-embedded and contained sufficient material for H&E staining and immunoassaying. Immunohistochemistry was performed according to the avidin-biotin complex (ABC) technique. Briefly, 60 sections were deparaffinized in xylene and rehydrated through graded ethanol washes. Ten minutes incubation in boiling citrate buffer (pH 6.0) was used for antigen retrieval. To block endogenous peroxidase, incubation with 3% hydrogen peroxidase in methanol for 5 minutes was performed. Non-specific binding was blocked with a 30 minute wash with 1% bovine serum albumin (BSA) in Tris-buffered saline (TBS, pH 7.6). The anti-PSMA mouse mAb (Novocastra, Newcastle, UK) was applied in dilution 1:50 in 30 specimens, and incubated overnight at 4°C. Anti-factor VIII mouse mAb [16] (DAKO, Ontario, Canada), in dilution 1:100, was also used in 30 sequencial slides of the same specimens to demonstrate the correct location of the endothelial cells. Following this, the slides were incubated with rabbit anti-mouse secondary mAb E0354 (diluted 1:500; DAKO, Ontario, Canada) for 30 minutes at 37°C. Sections were then incubated with horseradish peroxidase-conjugated ABC complex (DAKO, Ontario, Canada) for 30 minutes at 37°C. Immunostaining was visualized using 3-amino-9-ethylcarbazole (AEC) chromogen (DAKO, Ontario, Canada). Finally, the slides were counterstained with Giu-II haematoxylin and cover-slipped. Prostatic hyperplasia and tonsil were used as positive controls. Negative control sections were incubated with non-immune serum (0.1% BSA in TRIS) instead of the primary antibody.

Samples were classified in two categories: negative (if none of the endothelial cells displayed immunostaining) and positive (if any endothelial cell displayed distinctive immunostaining, irrespective of the staining intensity). In addition, for each specimen the percentage of cells stained (less than 5% = 0; 6% to 25% = 1; 26% to 50% = 2; 51% to 75% = 3; and greater than 75% = 4) was determined. This criterion has been previously used [17].

## Results

**Figure 1:**
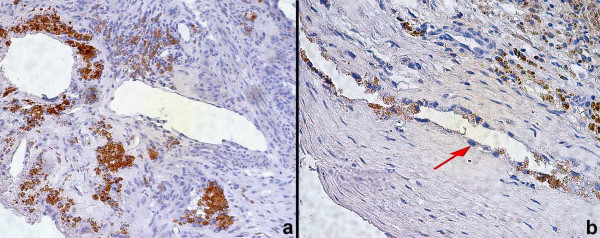
Endothelial cells of choroidal neovascular membrane stained negative to PSMA, 200× (a) and 400× (b, arrow).

**Figure 2:**
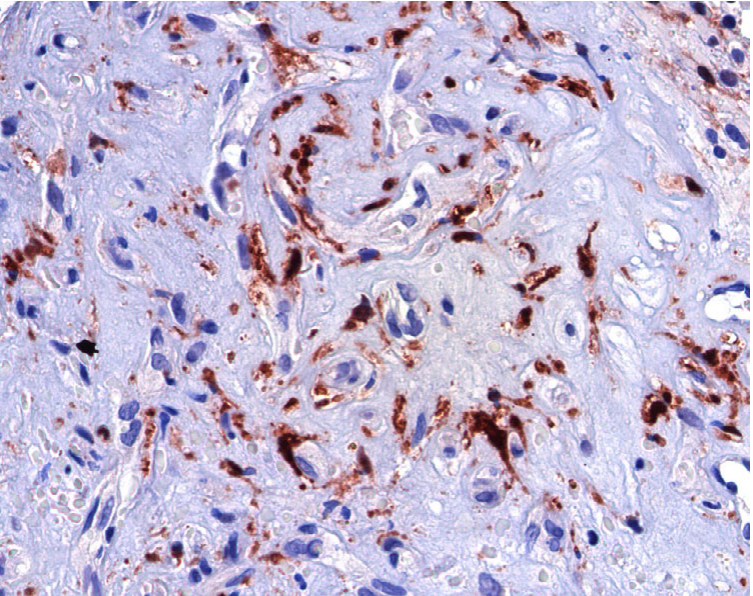
Endothelial cells of choroidal neovascular membrane stained positive to factor-VIII, 400×.

**Figure 3:**
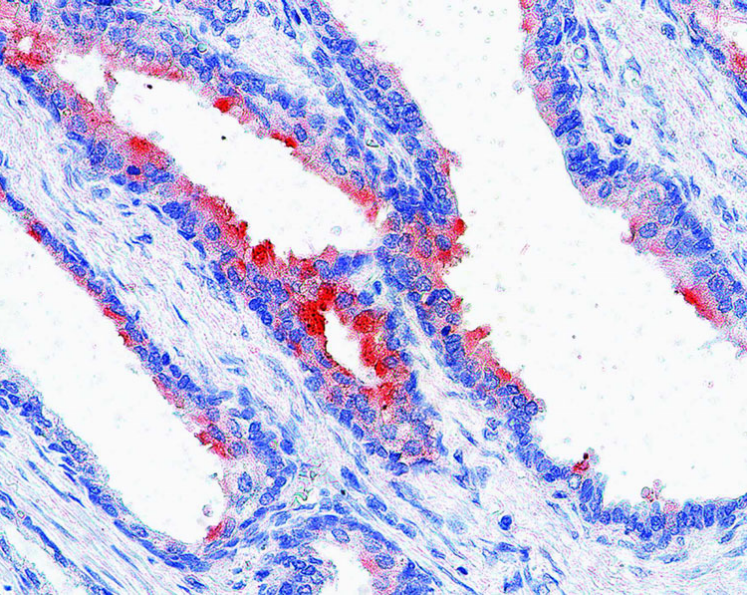
Secretory-acinar epithelium of a hyperplastic prostate stained positive to PSMA, 400×.

To evaluate the PSMA expression, immunohistochemical analyses with anti-PSMA and anti-factor VIII mAbs were performed in 30 cases of CNVM. The endothelial cells of the angiogenic microvessels of all the cases demonstrated negative staining to PSMA (Figure 1a,1b) while factor VIII was expressed in the endothelial cells of all CNVM sequencial slides (Figure 2). Seventy-five percent of the prostatic secretory-acinar epithelium of the prostatic hyperplasia specimen (Figure 3) used as positive control, stained positive. This confirmed that the immunohistochemical technique was correctly performed.

## Discussion

The PSMA protein's name may be misleading since its expression is not confined exclusively to the prostate. It has also been reported in select benign non-prostatic tissues such as duodenal columnar epithelium, proximal renal tubular epithelium, colonic ganglion cells and benign breast epithelium [9,13-15,18].

**Figure 4:**
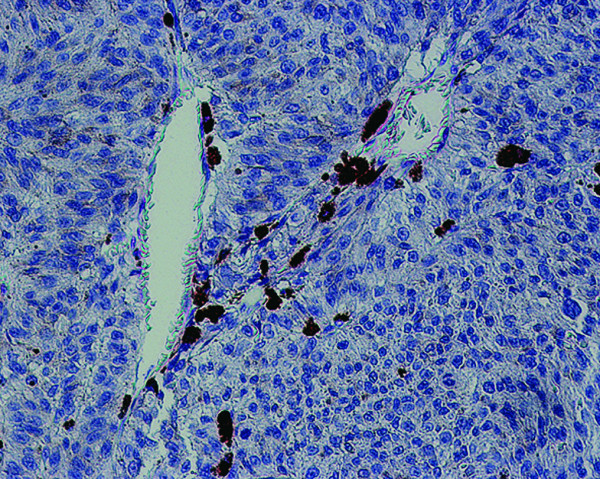
Endothelial cell of neovascularization related to uveal melanoma and malignant melanocytes stained negative to PSMA.

Several immunohistochemical studies have examined expression of PSMA in non-prostatic primary tumor neovasculature. Silver et al [14] demonstrated the expression of PSMA in endothelial cells in a subset of tumors including renal cell carcinoma, transitional cell carcinoma of the urinary bladder, and colonic adenocarcinoma. More recently, Chang et al [19] examined multiple anti-PSMA antibodies and showed that each antibody consistently bound the tumor-associated neovasculature in cases of testicular embryonal carcinoma, neuroendocrine carcinoma, malignant skin melanoma, pancreatic duct carcinoma, non-small cell lung carcinoma, soft tissue sarcoma, and breast carcinoma. In our laboratory, a previous study (unpublished data) demonstrated that PSMA is not expressed in neovasculature associated to uveal melanoma or in malignant uveal melanocytes (Figure 4).

Interestingly, neither the vascular endothelial cells of benign tissues nor the neoplastic cells of vascular tumors expressed PSMA [19]. These results suggest that endothelial cell PSMA expression may be restricted to tumor-associated neovasculature and is probably stimulated by some tumor-secreted angiogenic factors [19]. RT-PCR and *in situ *hybridization analyses have also demonstrated that PSMA protein is produced by endothelial cells of tumor-associated neovasculature instead of being sequestered from the serum or from the surrounding stromal cells [18]. However, the hypothesis that PSMA is specifically expressed by endothelial cells from tumor-related neovasculature should not be unconditionally accepted since PSMA expression in benign neovasculature has not yet been established.

The purpose of this study was to characterize the PSMA expression in 30 cases of benign neovasculature, while confirming the idea that endothelial cell PSMA expression is restricted to tumor-associated neovasculature. Factor VIII, a specific endothelial marker, was used to demonstrate the exact location of the endothelial cells [16]. Furthermore, if PSMA is present in CNVM, it may represent a potential target for antineovasculature-based therapy in cases of AMD.

## Conclusion

Since none of the 30 cases of CNVM expressed PSMA, this protein may not represent a new target for antineovasculature-based therapy in cases of AMD. Further studies will need to be done to validate this hypothesis. Based on the results of the present study, the hypothesis that endothelial cell PSMA expression is restricted to tumor-associated neovasculature is plausible. However, the PSMA expression in other benign neovasculature tissue, such as granulation tissue, remains to be established.

## Competing interests

The author(s) declare that they have no competing interests.

## References

[B1] Cruickshanks KJ, Hamman RF, Klein R, Nondahl DM, Shetterly SM (1997). The prevalence of age-related maculopathy by geographic region and ethnicity. The Colorado-Wisconsin Study of Age-Related Maculopathy. Arch Ophthalmol.

[B2] Klein R, Klein BE, Linton KL (1992). Prevalence of age-related maculopathy. The Beaver Dam Eye Study. Ophthalmology.

[B3] Maberley DA, Hollands H, Chuo J, Tam G, Konkal J, Roesch M, Veselinovic A, Witzigmann M, Bassett K (2006). The prevalence of low vision and blindness in Canada. Eye.

[B4] Mitchell P, Smith W, Attebo K, Wang JJ (1995). Prevalence of age-related maculopathy in Australia. The Blue Mountains Eye Study. Ophthalmology.

[B5] Oshima Y, Ishibashi T, Murata T, Tahara Y, Kiyohara Y, Kubota T (2001). Prevalence of age related maculopathy in a representative Japanese population: the Hisayama study. Br J Ophthalmol.

[B6] Ambati J, Ambati BK, Yoo SH, Ianchulev S, Adamis AP (2003). Age-related macular degeneration: etiology, pathogenesis, and therapeutic strategies. Surv Ophthalmol.

[B7] Grossniklaus HE, Green WR (2004). Choroidal neovascularization. Am J Ophthalmol.

[B8] Heriot WJ, Henkind P, Bellhorn RW, Burns MS (1984). Choroidal neovascularization can digest Bruch's membrane. A prior break is not essential. Ophthalmology.

[B9] Horoszewicz JS, Kawinski E, Murphy GP (1987). Monoclonal antibodies to a new antigenic marker in epithelial prostatic cells and serum of prostatic cancer patients. Anticancer Res.

[B10] Israeli RS, Powell CT, Fair WR, Heston WD (1993). Molecular cloning of a complementary DNA encoding a prostate-specific membrane antigen. Cancer Res.

[B11] Carter RE, Feldman AR, Coyle JT (1996). Prostate-specific membrane antigen is a hydrolase with substrate and pharmacologic characteristics of a neuropeptidase. Proc Natl Acad Sci U S A.

[B12] Pinto JT, Suffoletto BP, Berzin TM, Qiao CH, Lin S, Tong WP, May F, Mukherjee B, Heston WD (1996). Prostate-specific membrane antigen: a novel folate hydrolase in human prostatic carcinoma cells. Clin Cancer Res.

[B13] Lopes AD, Davis WL, Rosenstraus MJ, Uveges AJ, Gilman SC (1990). Immunohistochemical and pharmacokinetic characterization of the site-specific immunoconjugate CYT-356 derived from antiprostate monoclonal antibody 7E11-C5. Cancer Res.

[B14] Silver DA, Pellicer I, Fair WR, Heston WD, Cordon-Cardo C (1997). Prostate-specific membrane antigen expression in normal and malignant human tissues. Clin Cancer Res.

[B15] Liu H, Moy P, Kim S, Xia Y, Rajasekaran A, Navarro V, Knudsen B, Bander NH (1997). Monoclonal antibodies to the extracellular domain of prostate-specific membrane antigen also react with tumor vascular endothelium. Cancer Res.

[B16] Grossniklaus HE, Ling JX, Wallace TM, Dithmar S, Lawson DH, Cohen C, Elner VM, Elner SG, Sternberg PJ (2002). Macrophage and retinal pigment epithelium expression of angiogenic cytokines in choroidal neovascularization. Mol Vis.

[B17] Chang SS, Reuter VE, Heston WD, Gaudin PB (2001). Comparison of anti-prostate-specific membrane antigen antibodies and other immunomarkers in metastatic prostate carcinoma. Urology.

[B18] Chang SS, O'Keefe DS, Bacich DJ, Reuter VE, Heston WD, Gaudin PB (1999). Prostate-specific membrane antigen is produced in tumor-associated neovasculature. Clin Cancer Res.

[B19] Chang SS, Reuter VE, Heston WD, Bander NH, Grauer LS, Gaudin PB (1999). Five different anti-prostate-specific membrane antigen (PSMA) antibodies confirm PSMA expression in tumor-associated neovasculature. Cancer Res.

